# Professor Adriana Fiorentini: 1/11/1926–29/2/2016

**DOI:** 10.1177/2041669516653543

**Published:** 2016-06-27

Adriana Fiorentini was for many decades a pillar of the CNR Institute for Neurophysiology (then Neuroscience). On February 29, 2016, she passed away peacefully in her sleep, with a smile. A whole generation of vision scientists remembers her with love and respect. Her wisdom, intelligence, dedication to science, enthusiasm for research, and love of knowledge set an example to us all. She was a role model of respect, generosity, patience, collaboration, and true humility: discrete and reserved, always there for her students and colleagues.
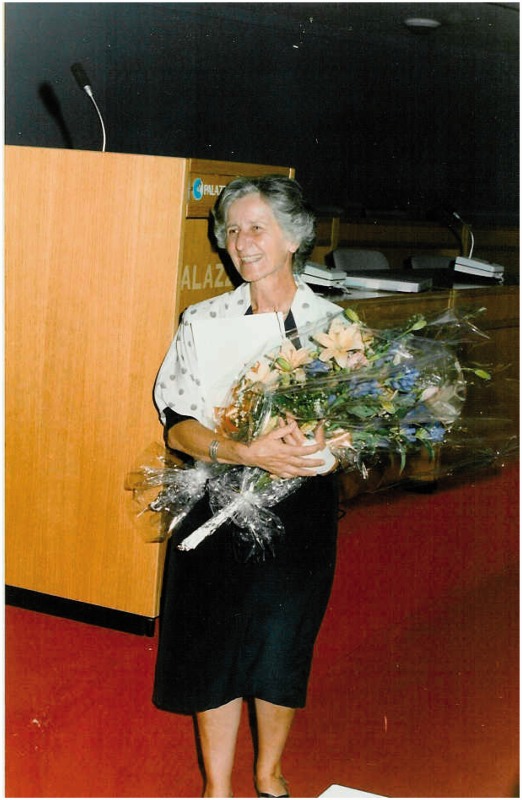


Adriana at ECVP 1992, Pisa, organized in her honor.

Born and schooled in Milan, she graduated in Physics in 1948 at the University of Florence, studying optics under Professor Giuliano Toraldo di Francia at the National Institute of Optics in Arcetri. During her studies she noticed a strange contrast phenomenon, which she correctly interpreted as the visual illusion known as “Mach Bands”; this observation was fundamental in shifting her research interests toward visual perception. In her early postgraduate years at Arcetri (1948–1968), Adriana published many innovative studies on physiological optics and perception, which are cited to this day. To mention just two: the seminal demonstration of human receptive fields (or “perceptive fields”) defined by antagonistic center-surround interactions; and her first (but not last) *Nature* paper (with Donald MacKay of Keele University) demonstrating a neural (VEP) correlate of a perceptual phenomenon.

Adriana wrote many book chapters, in Italian and English, on the perception of contrast and of brightness and lightness. She trained a generation of optometrists at Vinci, using her simple and clear textbook *Occhi e Occhiali* (Eyes and Glasses). It is not infrequent even now to enter an optometrist shop to discover that the owner was trained by Adriana and remembers her well with awe and affection.

In 1966, Adriana was encouraged by Professor Giuseppe Moruzzi to join forces with the young Lamberto Maffei at the CNR Institute of Neurophysiology. Working together, Lamberto and Adriana studied vision by (in the words of Maffei) “posing the same questions to human perception and mammalian single cortical cells,” attempting to uncover, where possible, the neural substrate of visual perception. This approach was extremely innovative in its day, and produced dozens of important papers that remain fundamental today.
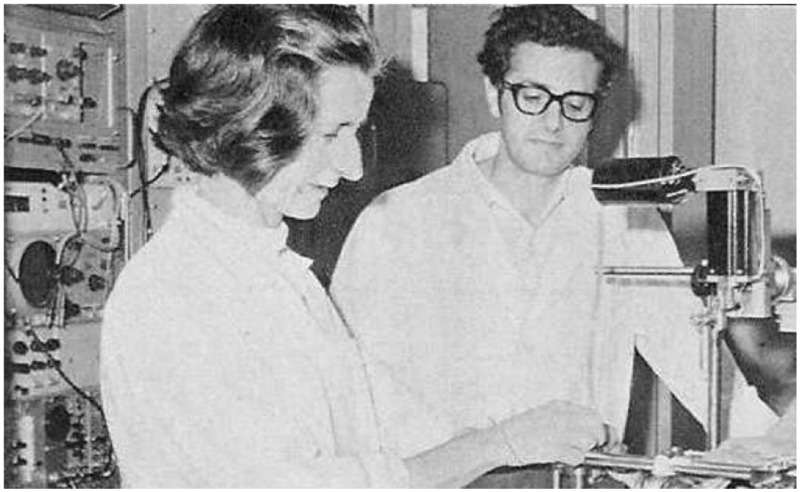
Adriana with the young Lamberto Maffei, at work in Pisa (circa 1975).

Adriana also pioneered the use of electrophysiological techniques to study visual development in humans and animals. This led to the first measurements of the development of infant contrast sensitivity, highlighted in all modern textbooks, and much fundamental information about the development of spatial and temporal neural properties. Another major contribution, still at the forefront of vision research to this day, is the demonstration of feature-selective perceptual-learning. We still remember her expression when the effects of learning disappeared on rotating the stimulus: but were magically restored by counter-rotation of her head. She often used this as an example for us to keep our eyes and our minds wide open.

Adriana was fundamental in forging a community of visual scientists in Europe, helping with the organization of the first European Conference of Visual Perception, and later organizing one herself in *Il Ciocco*, Garfagna, Tuscany. She was on the editorial board of *Perception* for many years, and on the editorial boards of *Vision Research*, *Brain and Behavioural Research*, *Clinical Vision Sciences*, and *Optica Acta.*

Throughout her years in science, Adriana was an excellent mentor to us students. She taught us to think clearly and analytically, to experiment rigorously and, most importantly, she taught dedication to our vocation by example. She was always available to discuss scientific ideas, and also life issues, actively participating in both our scientific and personal growth. During “journal club” discussion (usually over tea and one of her home-made cakes), she discussed not only the science of each paper, but joyfully related pleasant and amusing anecdotes of the scientists behind the science and their laboratories. This for us was fundamental, creating a visual map in our brains of the major international vision labs around the world.

Her interests were wide, embracing music, art, and religion, as well as the popularization of science. With Lamberto Maffei, she wrote *Arte e Cervello* (Art and Brain), a beautiful little book about the nature of visual perception and visual language, which was met with great success and received many prizes.

We remember many things about Adriana: that we always stole the biscuits she left in her drawer, and she pretended not to notice; Adriana’s amazing apple strudel and chocolate cake (*La Tenerina*) for which we have autographed recipes; Adriana making afternoon tea, which facilitated fantastic discussions about science; Adriana, elegant as always, in fancy dress disguised as Mach Bands; Adriana giving half of her salary to one of us who had been robbed of her first paycheck.

In the end, they are these little things that one remembers about a person, even of a great scientist: the generosity and willingness to listen; the sincerity, the energy and complexity of the person; and the genuine humility. These things remain.

She was a mentor and close friend to all of us: we will miss her.

Donatella Spinelli, Department of Human Movement, Social and Health Sciences, University of Rome, ‘Foro Italico’ University of Rome

Nicoletta Berardi, Department of Neuroscience, Psychology, Pharmacology and Child Health, University of Florence

Concetta Morrone, Department of Translational Research on New Technologies in Medicines and Surgery, University of Pisa

